# Metabolic alteration of circulating steroid hormones in women with gestational diabetes mellitus and the related risk factors

**DOI:** 10.3389/fendo.2023.1196935

**Published:** 2023-06-15

**Authors:** Na Yang, Wei Zhang, Cheng Ji, Jiajia Ge, Xiaoli Zhang, Meijuan Li, Min Wang, Tianqi Zhang, Jun He, Huaijun Zhu

**Affiliations:** ^1^ Department of Pharmacy, Nanjing Drum Tower Hospital, Affiliated Hospital of Medical School, Nanjing University, Nanjing, Jiangsu, China; ^2^ Nanjing Drum Tower Hospital Clinical College of Nanjing University of Chinese Medicine, Nanjing, Jiangsu, China; ^3^ Nanjing Qlife Medical Technology Co., Ltd, Nanjing, Jiangsu, China

**Keywords:** steroid hormones, gestational diabetes, UPLC-MS/MS, estrogens, corticosteroids, progestins

## Abstract

**Background:**

Abnormally changed steroid hormones during pregnancy are closely related to the pathological process of gestational diabetes mellitus (GDM). Our aim was to systematically profile the metabolic alteration of circulating steroid hormones in GDM women and screen for risk factors.

**Methods:**

This study was a case-control study with data measured from 40 GDM women and 70 healthy pregnant women during their 24-28 gestational weeks. 36 kinds of steroid hormones, including 3 kinds of corticosteroids, 2 kinds of progestins, 5 kinds of androgens and 26 kinds of downstream estrogens in serum were systematically measured using a combined sensitive UPLC-MS/MS method. The flux of different metabolic pathways of steroid hormones was analyzed. Logistic regression and ROC curve model analyses were performed to identify potential steroid markers closely associated with GDM development.

**Results:**

Serum corticosteroids, progestins and almost all the estrogen metabolites via 16-pathway from parent estrogens were higher in GDM women compared with healthy controls. Most of the estrogen metabolites via 4-pathway and more than half of the metabolites via 2-pathway were not significantly different. 16α-hydroxyestrone (16OHE1), estrone-glucuronide/sulfate (E1-G/S) and the ratio of total 2-pathway estrogens to total estrogens were screened as three indicators closely related to the risk of GDM development. The adjusted odds ratios of GDM for the highest quartile compared with the lowest were 72.22 (95% CI 11.27-462.71, *P*_trend_ *<*0.001) for 16OHE1 and 6.28 (95% CI 1.74-22.71, *P*_trend_ *<*0.05) for E1-G/S. The ratio of 2-pathway estrogens to total estrogens was negatively associated with the risk of GDM.

**Conclusion:**

The whole metabolic flux from cholesterol to downstream steroid hormones increased in GDM condition. The most significant changes were observed in the 16-pathway metabolism of estrogens, rather than the 2- or 4-pathway or other types of steroid hormones. 16OHE1 may be a strong marker associated with the risk for GDM.

## 1 Introduction

Gestational diabetes mellitus (GDM) is an idiopathic complication during pregnancy, characterized primarily by hyperglycemia first diagnosed during pregnancy. It is estimated that GDM affects more than 20 million live births worldwide (1). The prevalence of GDM varies from 1% to 28% in different countries and regions. Compared with European pregnant women, Asian pregnant women were found to have a higher incidence of GDM (2, 3). However, since the lack of uniformity in screening method and diagnosis criteria for GDM, the comparison on the prevalence of GDM between and within countries needs to be further discussed (4).

In terms of short-term effects, GDM can cause high blood pressure during pregnancy, high fetal weight and premature delivery (5). In the later long-term follow-up of women with GDM, they are at higher risk of developing type 2 diabetes (T2D) (6, 7), hypertension (8) and other cardiovascular events (9–12) than those without GDM. Moreover, emerging evidence tends to suggest that GDM also has profound influences on the health of offspring, whether short or long term. The newborns of GDM mothers may develop macrosomia, hypoglycemia, respiratory distress syndrome and some other severe complications (13). Henceforth, they may also risk long-term health problems including insulin resistance (IR), subsequent obesity, T2D, and increased neuropsychiatric morbidity (14).

At present, the pathogenesis of GDM is not very clear. IR, genetic susceptibility, metabolic disorders, and the interaction of complex factors are proved to be closely associated with GDM. In general, IR is considered to underlie the pathophysiology of GDM (15). IR may occur during pregnancy due to impairment of pancreatic islet β-cell function, which makes it fail to compensate for insulin. Current studies suggested other potential factors related to IR in pregnant women, including levels of steroid hormones, placental hormones, and inflammatory mediators, which function as antagonists to insulin (16).

**Figure 1:**
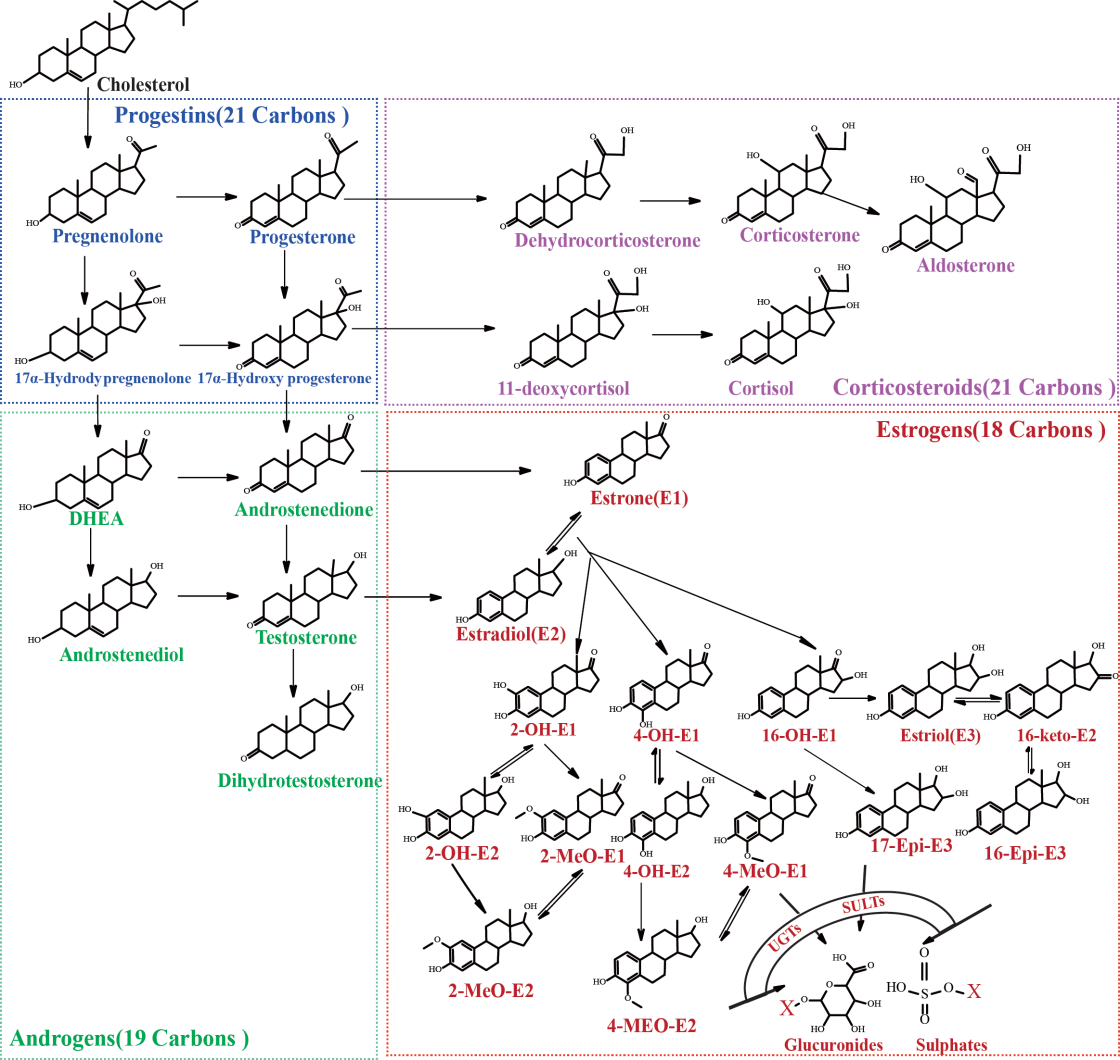
Metabolic network of steroid hormones.

As we all know, steroid hormones exert strong biological activities in our bodies via specific receptors. They could be divided into four classes according to the structures: estrogens, progestins, androgens and corticosteroids. These steroid hormones are closely linked in a metabolic network originated from cholesterol. Steroid hormones could affect insulin function in diverse aspects. For instance, progesterone, cortisol and estrogens were found to affect β-cell function or the sensitivity of peripheral tissues to insulin (17). CAR-mediated signaling pathway was confirmed to be involved in the influence of estrogens and progestins on IR (18). Progesterone may exert a toxic effect on pancreatic β-cells through an oxidative-stress-dependent mechanism that induces apoptosis (19). Hence, the abnormal changes of steroid hormone levels (e.g. testosterone, estradiol and progesterone, etc.) observed in women with GDM (19–24) are correlated with the occurrence and development of IR. Remarkably, most existing studies focused on several or several types of precursor steroid hormones, which are confirmed to have high biological activities and could be easily measured. However, in the metabolic network of steroid hormones, there are dozens of structural analogues with similar or opposite physiological activity (**Figure 1**). Changes of steroid levels are not only related to the activities of upstream or downstream enzymes but also to the levels of intermediates in the pathways. Focusing only on a few steroid hormones makes it difficult to trace the cause of metabolic changes or to reveal the potential mechanism of GDM pathogenesis. Precursor steroid hormones could be metabolized through reduction, oxidation, methylation, glucuronidation or other metabolic pathways *in vivo (* 25). Despite the relative low levels of some metabolites, they are proved to be involved in some physiological processes, such as fat disposition regulation and muscle mass promotion (26, 27). At present, there is still a lack of research on the overall metabolic network of steroid hormones in GDM pregnant women. The relationship between steroid metabolites and GDM is still unclear.

This study aims to systematically profile the changes of steroid metabolic network in pregnancy with GDM by employing a combined sensitive ultra-performance liquid chromatography-tandem mass spectrometry (UPLC-MS/MS). For the first time, the overall metabolic characteristics of steroid hormones in pregnant women with GDM were analyzed and summarized through quantifying 36 kinds of steroid hormones, including 3 kinds of corticosteroids, 2 kinds of progestins, 5 kinds of androgens and 26 kinds of downstream estrogens and their metabolites. This work contributes to further disclosing the correlation between the metabolic disorders of steroid hormones and the pathogenesis of GDM.

## 2 Materials and methods

### 2.1 Chemicals and materials

Estradiol (E2), estrone (E1) and estriol (E3) were purchased from China National Institutes for Food and Drug Control (Beijing, China). 2-Hydroxyestrone (2OHE1), 2-hydroxyestradiol (2OHE2), 2-methoxyestrone (2MeOE1), 2-methoxyestradiol (2MeOE2), 4-methoxyestrone (4MeOE1), 4-methoxyestradiol (4MeOE2), 16α-hydroxyestrone (16OHE1), 16-epiestriol (16EpiE3), 17-epiestriol (17EpiE3), aldosterone (ALD) and internal standards (deuterated steroid hormones) were purchased from TRC (Toronto, Canada). Progesterone (P4), 17α-hydroxyprogesterone (17α-OHP4), testosterone (T), dihydrotestosterone (DHT), androstenedione (AD), corticosterone (CORT) and cortisol (F) were purchased from Dr. Ehrenstorfer (Augsburg, Germany). Dehydroepiandrosterone (DHEA) was purchased from Cerilliant (Round Rock, TX, USA). Dehydroepiandrosterone sulfate (DHEA-S) was purchased from ISOscience (Ambler, PA, USA). 4-Hydroxyestrone (4OHE1), dansyl chloride, L-ascorbic acid, and β-glucuronidase/arylsulfatase were obtained from Sigma-Aldrich (St. Louis, MO). Methanol (HPLC grade) and formic acid were purchased from Merck (Darmstadt, Germany) or Fisher (Waltham, MA, USA). Deionized water was prepared using a Milli-Q system (Millipore, Milford, MA, USA). Acetone was obtained from Nanjing Chemical Reagent CO., LTD.

### 2.2 Study population and sample collection

This study was approved by the ethical committee of Drum Tower Hospital Affiliated to Medical School of Nanjing University (No.2021-021-01) and was registered to ChiCTR (identifier: ChiCTR2100048675). Written consent has been obtained from each patient or subject after full explanation of the purpose and nature of all procedures used. All women in this study had singleton pregnancies and underwent routine 75-g oral glucose tolerance test (OGTT) screening at 24-28 gestational weeks. GDM was diagnosed based on IADPSG guideline to meet one or more of the following diagnostic criteria (28): (1) fasting blood glucose≥5.1 mmol/L; (2) 1 h≥10.0 mmol/L; (3) 2 h≥8.5 mmol/L. The final study population was determined after excluding pregnant women with any of the following characteristics: (1) <18 years of age; (2) severe maternal or fetal illness (malignancy, decompensated liver disease, heart disease, hypertension, congenital anomaly, etc.); (3) pre-existing diabetes mellitus or overt diabetes; (4) polycystic ovarian syndrome; (5) presence of drug use that interferes with steroid metabolism (progestin drugs); (6) non-singleton pregnancies; (7) newborns diagnosed with congenital anomaly (heart disease, neurological disease, metabolic diseases, etc.) or missing health assessment information for the newborns. Finally, a total of 40 GDM women and 70 healthy pregnant women aged 26–42 were recruited for analysis.

### 2.3 Anthropometric and biochemical measurements

A detailed medical history including age, gestational age, height and other basic information was recorded for each participant. Maternal weight at 24-28 weeks of gestation was measured by the standard method. The pre-pregnancy weight was recorded according to patient statements. BMI value was calculated by dividing the weight (kilograms) by the square of height (meter^2^). Each participant underwent a 75-g OGTT after an overnight fast of 10–12 hours. Blood lipids (triglycerides (TG), total cholesterol (TC), high-density lipoprotein (HDL), and low-density lipoprotein (LDL) and liver enzymes, including aminotransferase (ALT), aspartate aminotransferase (AST), g-glutamyl transferase (GGT) and plasma glucose were measured by using a biochemical autoanalyzer (Beckman, CA, USA).

### 2.4 Quantification of the serum steroid hormones based on UPLC-MS/MS method

Steroid hormones in serum samples collected at the at 24-28 weeks of gestation were determined using a Waters UPLC I-Class system interfaced with a Xevo TQ-S triple quadrupole mass spectrometer (Waters Corp., Milford, MA, USA). This method validation was performed according to the Guidelines for Bio-analytical Method Validation. For the determination of estrogens, mass spectrometry was performed in positive mode and multiple reaction monitoring (MRM). Liquid chromatography separation was achieved on a Waters CORTECS C18 column (2.1 × 150 mm, 1.6 μm) at 40°C. The flow rate was 0.3 mL/min. The mobile phase consisted of solvent A, deionized water (containing 0.05% formic acid and 2 mM ammonium acetate) and solvent B, methanol. The elution gradient was as follows: 0.0-4.1min, isocratic 78% B; 4.1-4.2 min, linear gradient 78-81% B; 4.2-8.0 min, isocratic 81% B; 8.0-8.01min, linear gradient 81-78% B; 8.01-10.0min, isocratic 78% B. The total run time was 10.0 min. For the determination of corticosteroids, progestins and androgens, mass spectrometry was performed in a positive and negative ion switching mode and MRM. Liquid chromatography separation was achieved on a Phenomenex Kinetex XB-C18 (3.0 × 50 mm, 2.6 µm) at 40°C. The flow rate was 0.3 mL/min. The mobile phase consisted of solvent A, deionized water (containing 100 μM ammonium fluoride) and solvent B, methanol. The elution gradient was as follows: 40% B increased to 98% B from 0.0 min to 3.0 min, maintained for 0.5 min, retuned to initial conditions, and then re-equilibrated for 2.5 min. The total run time was 6.0 min.

Sample preparation procedure was performed as previously described (29, 30). Briefly, charcoal-stripped human serum with no detectable levels of any steroid hormones was used for preparation of calibration standards and quality control samples. Free estrogens, conjugated (-glucuronide/sulfate, -G/S) estrogen metabolites and other steroid hormones were directly or indirectly measured through preparation procedures with or without β-glucuronidase/sulfatase hydrolysis step. Serum samples and enzymatically hydrolyzed serum samples underwent liquid-liquid extraction with methyl tert-butyl ether. After extraction, the organic solvent portion was transferred and evaporated to dryness at 60°C under nitrogen gas. For the determination of estrogens, the dried samples then underwent the derivatization process using dansyl chloride. All residue samples were reconstituted with methanol and analyzed by UPLC-MS/MS.

### 2.5 Statistical analysis

The normality of values was analyzed by Shapiro-Wilk test. Values with normal distribution were summarized as the mean ± standard deviation (SD), and the corresponding significance was examined by using the Student’s t-test. Values with non-normality were summarized as the median (interquartile range, IQR), and significance was examined by Mann-Whitney U test. *P *< 0.05 was considered significant. Volcano graph was drawn by GraphPad Prism 8.0 to screen for the obvious changes of serum steroid hormones in GDM women compared to healthy pregnant women. A mixed-effects Least Absolute Shrinkage Selection Operator (LASSO) logistic regression, implemented by STATA/MP version 16.0, was used for multicollinearity elimination and variable selection. Receiver operating characteristic (ROC) curves were plotted to classify the performance of biomarkers. The most relevant steroid hormones are grouped into quartiles to determine the linear trend relationship between the independent variables and the prevalence of GDM.

## 3 Results

### 3.1 Establishment and method validation of UPLC–MS/MS analysis

**Figure 2:**
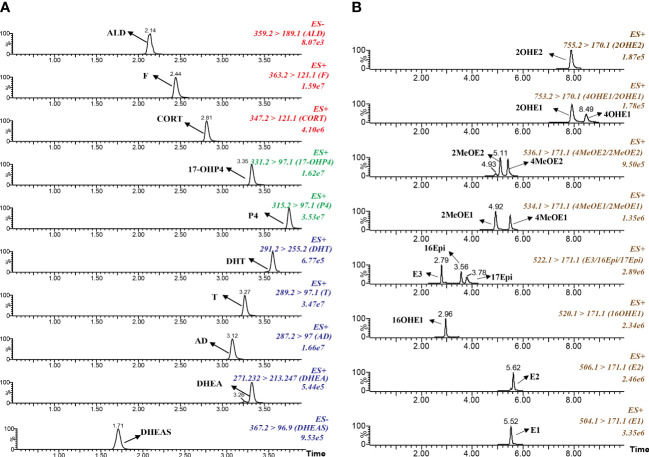
Typical chromatograms of multiple reaction monitoring for target steroid hormones. **(A)** Corticosteroids, progestins and androgens. **(B)** Estrogens and the metabolites.

A combined sensitive and rapid UPLC-MS/MS method was developed to profile the steroid network in GDM women. 23 kinds of steroid hormones, including 5 kinds of upstream C21 steroid hormones (corticosteroids and progestins), 5 kinds of C19 steroid hormones (androgens) and 13 kinds of downstream C18 steroid hormones (estrogens), were directly measured through the UPLC-MS/MS method. 13 kinds of conjugated Phase II metabolites were indirectly measured by the β-glucuronidase/sulfatase hydrolysis method. As shown in **Figure 2**, we optimized the elution gradient of chromatographic mobile phases to achieve rapid separation of multiple isomers. The peaks for each steroid appeared sharp and symmetrical. The total running time kept within 6 mins and 10 mins. This method validation was performed according to the guidelines for Bio-analytical Method Validation published by the US FDA and fulfilled the acceptance criteria, as shown in **Supplementary Tables S1–4**. The linear range of the measured steroid hormones was as follows: estrogens (0.001-2 ng/mL), T (0.05-10 ng/mL), DHT (0.025-5 ng/mL), 17α-OHP4(0.05-10 ng/mL), P4 (0.05-10 ng/mL), CORT (0.1-20 ng/mL), DHEA (0.1-20 ng/mL), AD (0.05-10 ng/mL), F (1-200 ng/mL), ALD (0.01-2 ng/mL), DHEA-S (50-10000 ng/mL).

### 3.2 Monitoring parameters

A total of 110 pregnant women participated in this study, including 40 GDM women and 70 healthy pregnant women. The baseline characteristics of the GDM group and corresponding controls are shown in **Table 1**. There was no statistical significance in age and weight between GDM and healthy pregnant women. GDM women had higher BMI values and higher levels of serum fasting and post-load glucose, TG and HDL compared with healthy pregnant women. The liver function index and other serum lipids were comparable between the two groups.

**Table 1:** Clinical characteristics of GDM cases.

	GDM (n = 40)	Control (n = 70)
Age (year)	30.5 (28.5-34.5)	29 (27-32)
Pre-pregnancy weight (kg)	60.7 ± 9.9	57.8 ± 10.6
Pre-pregnancy BMI (kg/m2)	23.7 ± 3.8	22.2 ± 3.7
Pregnancy weight (kg)	69.62 ± 10.46	66.34 ± 9.56
Pregnancy BMI (kg/m2)	27.1 ± 3.74 *	25.55 ± 3.15
SBP (mmHg)	116.2 ± 7.4	113.1 ± 8.8
DBP (mmHg)	73.8 ± 6.4	70.9 ± 8.5
ALT(IU/L)	16.95 (10.95-24.54)	18.65 (11.2-29.6)
AST(IU/L)	16.7 (14.1-20.67)	19 (14.3-23.2)
GGT(IU/L)	17.15 (10.75-24.87)	13.95 (11.3-18.1)
TG(mmol/L)	2.47 (1.93-3.35)**	1.85 (1.65-2.29)
TC(mmol/L)	5.69 ± 1.14	5.58 ± 0.97
HDL(mmol/L)	1.96 (1.63-2.3)*	2.12 (1.89-2.5)
LDL(mmol/L)	2.70 (2.32-3.34)	3.00 (2.46-3.43)
FPG (mmol/L)	4.67 (4.31-5.30)**	4.13 (4.12-4.56)
1hPG (mmol/L)	10.4 (9.55-10.77)**	7.6 (6.6-8.3)
2hPG (mmol/L)	8.5 (7.2-9.37)**	6.15 (5.6-7)

The normality of values was analyzed by Shapiro-Wilk test. Values with normal distribution were expressed as Mean ± SD. Values with non-normality were expressed as median and interquartile range (Q1~Q3). BMI: body mass index; SBP: systolic blood pressure; DBP: diastolic blood pressure; FPG: fasting plasma glucose; 1hPG:1-hour plasma glucose; 2hPG:2-hour plasma glucose. Pregnancy weight was measured at 24-28 gestational weeks.

* p <0.05, ** p <0.01, compared with control group.

### 3.3 Profiling of serum steroid hormones in women with GDM

The levels of 36 steroid hormones measured by UPLC-MS/MS are presented in **Table 2**. Briefly, in addition to androgens, there was a statistically significant increase in serum corticosteroid, progestin and some downstream estrogen levels of GDM women, compared with the healthy. The levels of serum androgens are roughly comparable. Changes in serum concentrations of progestins and corticosteroids were relatively mild between GDM and healthy women, except for 17-OHP4 (*p*<0.05, FC 1.6), an active intermediate derived from P4 via 17-hydroxylase.

**Table 2:** The concentrations of serum steroids and metabolites in GDM women and healthy pregnant women.

	Control (n = 70)	GDM (n = 40)	Trend	FC	*p* value
Corticosteroids					
ALD	0.28 (0.15-0.40)	0.38 (0.20-0.53)	↑	1.36	<0.05
CORT	6.39 (3.98-8.25)	7.53 (5.00-10.72)	↑	1.18	<0.05
F	212.36 (177.95-266.30)	253.48 (213.61-290.24)	↑	1.19	<0.01
Progestins					
P4	52.58 (44.26-63.26)	66.99 (49.68-86.88)	↑	1.27	<0.001
17-OHP4	2.19 (1.89-2.63)	3.50 (2.07-3.02)	↑	1.60	<0.05
Androgens					
T	0.77 (0.54-1.18)	0.90 (0.53-1.23)	–	–	0.73
AD	1.60 (1.14-2.34)	1.87 (1.26-2.48)	–	–	0.445
DHT	0.19 (0.14-0.22)	0.17 (0.13-0.24)	–	–	0.504
DHEA	1.15 (0.79-1.61)	1.03 (0.73-1.64)	–	–	0.543
DHEAS	1180.21 (864.60-1631.76)	1297.71 (835.10-1645.34)	–	–	0.931
Estrogens					
Parent estrogens and -G/S metabolites					
E1	1.52 (0.83-2.42)	1.82 (1.32-2.95)	↑	1.20	<0.05
E1-G/S	11.82 (9.11-13.83)	14.43 (12.34-16.62)	↑	1.22	<0.001
E2	6.55 (5.39-7.78)	8.91 (6.55-11.58)	↑	1.36	<0.001
E2-G/S	0.00 (0.00-0.00)	0.00 (0.00-0.00)	–	–	
16-Hydroxylation pathway estrogens and -G/S metabolites					
E3	3.54 (2.89-4.36)	5.99 (4.26-7.77)	↑	1.69	<0.001
E3-G/S	15.87 (10.69-21.39)	24.94 (15.96-37.42)	↑	1.57	<0.001
16OHE1	0.35 (0.28-0.41)	0.58 (0.47-0.71)	↑	1.66	<0.001
16OHE1-G/S	9.78 (7.77-14.19)	17.48 (12.89-26.45)	↑	1.79	<0.001
16EpiE3	0.064 (0.052-0.077)	0.100 (0.082-0.134)	↑	1.56	<0.001
16EpiE3-G/S	0.43 (0.32-0.60)	0.61 (0.49-0.96)	↑	1.42	<0.001
17EpiE3	0.030 (0.025-0.037)	0.044 (0.03-0.053)	↑	1.47	<0.001
17EpiE3-G/S	0.18 (0.10-0.40)	0.28 (0.13-0.48)	–	–	0.09
2-Hydroxylation pathway estrogens, methylated and -G/S metabolites					
2OHE1	0.0032 (0.0024-0.0043)	0.0051 (0.0033-0.0067)	↑	1.59	<0.01
2OHE1-G/S	0.51 (0.37-0.88)	0.49 (0.25-0.67)	–	–	0.297
2OHE2	0.0041 (0.0032-0.0052)	0.0064 (0.0043-0.0083)	↑	1.56	<0.001
2OHE2-G/S	0.030 (0.017-0.071)	0.026 (0.016-0.047)	–	–	0.359
2MeOE1	0.48 (0.28-1.42)	0.50 (0.34-0.90)	–	–	0.943
2MeOE1-G/S	0.00 (0.00-0.02)	0.00 (0.00-0.006)	–	–	
2MeOE2	0.58 (0.39-0.95)	0.80 (0.59-1.44)	↑	1.38	<0.01
2MeOE2-G/S	0.00 (0.00-0.00)	0.00 (0.00-0.00)	–	–	
4-Hydroxylation pathway estrogens, methylated and -G/S metabolites					
4OHE1	0.0024 (0.00170-0.0028)	0.0033 (0.0022-0.0042)	↑	1.38	<0.01
4OHE1-G/S	0.0121 (0.0059-0.0201)	0.0132 (0.0081-0.0276)	–	–	0.275
4MeOE1	0.0017 (0.0016-0.0018)	0.0016 (0.001-0.0018)	–	–	0.08
4MeOE1-G/S	0.0064 (0.0042-0.0092)	0.0072 (0.0061-0.0086)	–	–	0.134
4MeOE2	0.00 (0.00-0.00)	0.00 (0.00-0.00)	–	–	
4MeOE2-G/S	0.0082 (0.0056-0.0126)	0.0086 (0.0067-0.0117)	–	–	0.765

FC, fold change.

Among all changed steroid hormones, estrogens showed relatively large changes. Among all kinds of estrogens measured, 3 kinds of conjugated metabolites, including E1-G/S, E3-G/S and 16OHE1-G/S, have absolute advantages in serum concentrations. The median concentrations of serum parent estrogens (E1, *p*<0.05; E2, *p*<0.001) and the conjugate metabolite (E1-G/S, *p*<0.001) in GDM women were higher than those in healthy women. E1-G/S was nearly 10 times higher in serum than its precursor E1. However, E2-G/S was not detected in serum samples. Interestingly, almost all metabolites via the 16-hydroxylation pathway from parent estrogens were higher in GDM women. Among these 16-hydroxylated metabolites, the median concentrations of 5 metabolites, including E3, E3-G/S, 16OHE1, 16OHE1-G/S, and 16EpiE3 (*p*<0.001), changed more than 1.5 times in the serum of GDM women compared with healthy women. In contrast, most estrogen metabolites via 4-hydroxylation pathway and more than half of the metabolites via 2-hydroxylation pathway were not significantly different in serum between the two groups. Besides, 2MeOE2-G/S and 4MeOE2 were not detected in all serum samples. 2MeOE1-G/S was not detected in more than 80% of serum samples.

From the perspective of metabolic flux, there was a significantly higher flux of hydroxylation metabolism of estrogens (*p*<0.001, FC 1.55) in GDM women, and this elevation of hydroxylation was mainly contributed by 16- hydroxylation pathway, as shown in **Table 3**. No significant difference was observed in the total calculated concentration of metabolites via 2- or 4-hydroxylation pathways. In addition, the metabolic ratios of 2- and 4-pathways to total estrogens decreased significantly in GDM women.

**Table 3:** Analysis on the tendency of metabolic pathway.

	Control (n = 70)	GDM (n = 40)	Trend	FC	*p* value
**Total estrogens**	88.31 (74.24-106.84)	133.58 (107.05-172.69)	↑	1.51	<0.001
**Hydroxylation pathway**	33.80 (27.24-43.69)	52.34 (39.89-74.79)	↑	1.55	<0.001
*16-pathway*	31.25 (23.42-41.84)	50.49 (37.83-72.32)	↑	1.62	<0.001
*2- pathway*	1.76 (1.20-3.45)	2.13 (1.37-2.95)	–	–	0.646
*4-pathway*	0.035 (0.024-0.047)	0.037 (0.028-0.053)	–	–	0.189
**Total conjugated estrogens**	39.23 (31.44-50.95)	59.82 (46.00-78.83)	↑	1.52	<0.001
**Total methylated estrogens**	1.15 (0.72-2.43)	1.54 (1.03-2.52)	–	–	0.133
Metabolic pathway ratios					
**Hydroxylation pathway: Total estrogens**	0.39 (0.36-0.42)	0.41(0.38-0.44)	↑	1.05	0.023
*16- pathway: Total estrogen*s	0.35 (0.32-0.40)	0.38 (0.35-0.42)	↑	1.09	0.008
*2- pathway: Total estrogen*s	0.021 (0.012-0.038)	0.016 (0.011-0.023)	↓	0.75	0.008
*4- pathway: Total estrogen*s	0.00034 (0.00027-0.00057)	0.00028 (0.00018-0.00044)	↓	0.83	0.036
**Methylated estrogens: Total estrogens**	0.013 (0.008-0.028)	0.013 (0.008-0.017)	–	–	0.102
**Methylated estrogens: Hydroxylation pathway**	0.70 (0.61-0.75)	0.75 (0.67-0.82)	↑	1.08	0.009
*2- Methylated: 2- pathway*	0.70 (0.61-0.75)	0.76 (0.68-0.82)	↑	1.08	0.005
*4- Methylation: 4-pathway*	0.54 (0.45-0.63)	0.51 (0.34-0.61)	–	–	0.409
**Conjugation: Total estrogens**	0.45 (0.43-0.47)	0.45 (0.43-0.47)	–	–	0.723

FC, fold change.

### 3.4 Correlation analysis reveal potential steroid markers associated with GDM

**Figure 3:**
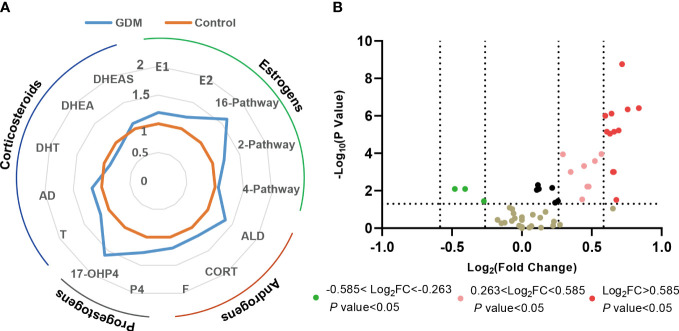
Changes of steroid metabolism in GDM women. **(A)** Phase diagrams showed the changes in different types of serum steroid hormones in GDM women compared with the health controls. **(B)** Volcano plot of serum steroid hormones in GDM women compared with the health controls. FC, fold change.

**Figure 4:**
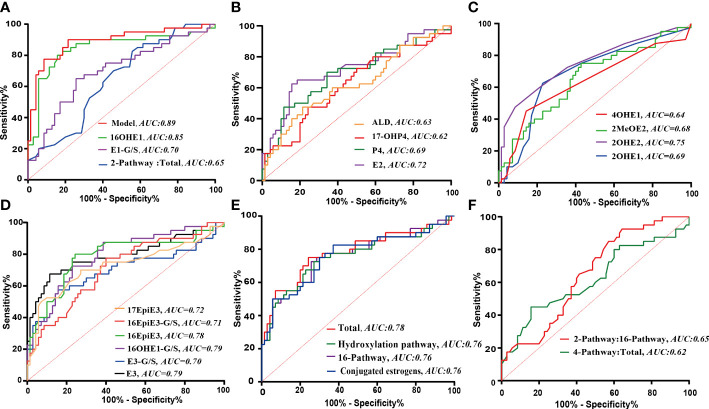
The ROC analyses of significantly changed indicators. **(A)** ROC curves of the combination model and the screened individual elements. **(B–F)** ROC curves of the steroid indicators with statistically significant change in GDM. B: Progestins, androgens and parent estrogens; C: Estrogens in 2- or 4- pathway; D: Estrogens in 16- pathway; E: Calculated pathway flux; F: Calculated pathway ratio.

As shown in **Figures 3**, **4**, a total of 23 kinds of steroid indicators were screened out according to Student’s t-test results and fold change values (*p*<0.05, FC>1.2). ROC analysis was further employed to preliminarily evaluate the diagnostic performance of serum metabolites in the discrimination of GDM from controls. The area under the curve (AUC) values of 9 directly measured steroid hormones (16OHE1, 16OHE1-G/S, E3, E1-G/S, E2, 16-EpiE3-G/S, 17-EpiE3, 16-epiE3 and 2OHE2) and 4 indirectly calculated indicators (total estrogens, conjugated estrogens, total hydroxylated estrogens, and total 16-hydroxylated estrogens) achieved good AUC values (>0.7), which indicated good diagnostic efficacy and close correlation. Among these indicators, 16OHE1 achieved the highest AUC value of 0.85. Based on all quantitative results, a combined model was established through LASSO logistic regression considering the basic biochemical characteristics, directly measured and indirectly calculated steroid indicators to eliminate the multicollinearity and find the specific markers associated with GDM. With parameter lambda of 0.05 obtained by cross-validation, we identified a diagnostic panel of 5 elements, including BMI, TG, 16OHE1, E1-G/S and the ratio of total 2-pathway estrogens to total estrogens. The equation Logit (P) = 0.172*BMI+0.438*TG+0.104*E1-G/S+7.381*16OHE1-29.131* ratio (2-pathway: total)-10.172. The AUC-ROC of the model was 0.89, which indicated that the screened markers are closely related to the development of GDM.

To further explore the correlation between the screened steroid hormones and the risk of GDM, the concentrations of serum 16OHE1, E1-G/S and the ratio of 2-pathway metabolites to total estrogens were stratified into quartiles to perform binary logistic regression analysis, as shown in **Table 4**. It was observed that 16OHE1 and E1-G/S were significantly positively correlated with GDM risk. This association was also enhanced in the age, BMI and TG–adjusted models. The age, BMI and TG adjusted odds ratios (ORs) of GDM for the highest quartile compared with the lowest were 72.22 (95% CI 11.27-462.71, *P*_trend_ *<*0.001) for 16OHE1 and 6.28 (95% CI 1.74-22.71, *P*_trend_< 0.05) for E1-G/S. Besides, the ratio of total 2-pathway estrogens to total estrogens was negatively associated with the risk for GDM, whether with or without adjustment for potential confounders. After adjustment by potential confounders, the negative association between the ratio of total 2-pathway estrogens to total estrogens and the risk for GDM was not significant (adjusted OR for the highest versus the lowest quartile, 0.65; *P*_trend_= 0.108).

**Table 4:** Multivariate-adjusted association of the selected indicators and the risk of GDM.

Characteristic	OR (95%CI)				*P*_trend_
	Quartile 1	Quartile 2	Quartile 3	Quartile 4	
**16OHE1**	≤0.309	0.310-0.404	0.405-0.560	≥0.561	
Crude OR	1.00 (reference)	0.67 (0.10-4.34)	6.25 (1.52-25.66)	47.92 (9.67-237.45)	<0.001
Adjusted OR*	1.00 (reference)	0.68 (0.06-3.48)	6.89 (1.42-33.30)	72.22 (11.27-462.71)	<0.001
**E1-G/S**	≤9.510	9.511-12.779	12.780-15.338	≥15.339	
Crude OR	1.00 (reference)	1.05 (0.29-3.77)	2.37 (0.73-7.71)	6.23 (1.89-20.57)	<0.01
Adjusted OR*	1.00 (reference)	1.14 (0.30-4.36)	1.66 (0.45-6.11)	6.28 (1.74-22.71)	<0.05
**2-pathway: total**	≤0.012	0.013-0.02	0.021-0.032	≥0.033	
Crude OR	1.00 (reference)	1.67 (0.57-4.84)	0.53 (0.18-1.62)	0.30 (0.09-1.03)	<0.05
Adjusted OR*	1.00 (reference)	2.80 (0.86-9.12)	1.02 (0.27-3.83)	0.65 (0.17-2.52)	0.108

*Conditional logistic regression (matching variables: age, BMI and triglycerides). OR, odds ratio.

## 4 Discussion

So far, various factors related to the pathogenesis and development of GDM have been investigated. Although the occurrence of IR in pregnant women may be closely related to the levels of steroid hormones, there is no data available on the alteration of the whole steroid metabolic network during the pregnancy process of GDM. Limited studies reported several kinds of serum steroid hormones in GDM patients based on immunoassay with lower specificity. Due to the different commercial kits, the results vary across laboratories (31–33) and the types of analyzed steroid hormones are limited. Hence, some potentially important steroid hormones have not been fully explored.

Generally, large amounts of progestogens and estrogens are produced in the mother’s body to adapt to the homeostasis during pregnancy (34). Claudio Villarroel et al. (24) studied 24 GDM cases and 24 control women during the second half of pregnancy and found that GDM pregnant women had lower E1 and E2 serum levels but a higher T level. Junguk Hur et al. (35) showed that an early second trimester serum unconjugated E3>95th percentile of the values generated from the overall screen population was associated with an increased risk for GDM (OR 2.05, *p*<0.001). However, conjugated E3 was not detected in the study. As a supplement, our results indicated that both conjugated and unconjugated E3 levels in GDM women were significantly higher than those in the healthy pregnant controls. Consistent with the previous findings that GDM women had higher P4 levels (36), our results demonstrated that GDM women also had significantly higher levels of 17-OHP4 (an active metabolite of P4) compared to the healthy pregnancy controls.

Abnormal levels of steroid hormones have been reported to modulate pancreatic function and susceptibility to develop IR (37). Vejrazkova D et al. (38) proposed that the effect of progesterone was probably related to the increase of IR due to a reduction of GLUT4 expression. Another study provided new insights that progesterone can be toxic to β-cells through oxidative stress mechanism that induces apoptosis (19). Besides, estradiol was proved to exert effects on carbohydrate metabolism and showed bidirectional regulation effects in many studies. Estradiol was found to prevent IR in female mice (39). However, estradiol at a high level could suppress GLUT4 gene expression (40), which may contribute to the process of IR. For a long time, the research on the relationship between estrogens and IR is limited and inconclusive.

Our results suggested that the risk for GDM seemed to be significantly elevated with increased 16-pathway metabolism and decreased 2-pathway metabolism. It is worth mentioning that 16OHE1 may be a strong marker associated with the risk for GDM (adjusted OR for the highest versus the lowest quartile, 72.22; *P*_trend_ < 0.001). 16OHE1 is a metabolite derived from 16-hydroxylase metabolism of estrone and could exert potent biological estrogenic effects. Most studies focused on exploring the relationship between 16OHE1 and breast cancer or other gynecological cancer (41). The ratio of 2OHE1 to 16OHE1 is inversely correlated with the risk for breast and cervical cancer. Hence, 16OHE1 is traditionally considered to be a harmful estrogen metabolite, while 2-hydroxylation of estrogen is contrary. The potential mechanisms include that high level of 16OHE1 could exert a strong biological estrogenic effect, which may promote cytotoxicity and cause cell changes or apoptosis (42). To some extent, this viewpoint is consistent with our results. However, further studies are needed to clarify the relationship between 16OHE1 and the pathological process of GDM or the destruction of β cell function. Besides, the gene polymorphisms of CYP1A1 and CYP1B1 were reported to be involved in regulating the ratio of 2OHE1 to 16OHE1 *in vivo* (43, 44), and their correlation with the pathogenesis of GDM still needs further clarification.

As our results indicated, E1-G/S is closely related to the risk of GDM. E1-G/S represents the metabolites of glucuronidation and sulfation of E1. Previous studies revealed that UGT1A10 and SULT1E1 are key enzymes involved in E1 glucuronidation and sulfation, respectively (45, 46). UGT1A10 is an extrahepatic enzyme and is exclusively expressed in gastrointestinal tract, kidneys, etc. SULT1E1 has the highest affinity for E1, E2 and catechol estrogens and is expressed in lung, liver and kidneys. The expression of SULT1E1 is highly related to breast and endometrial cancers since it could inactivate estrogens *in vivo*. E1-S was supposed to give a better indication of the extent of aromatase inhibition than E1 or E2 (47). Besides, genetic polymorphisms of SULT1E1 and UGT1A10 (e.g., *SULT1E1* rs11569705, rs11569705; *UGT1A10* rs2741049) have been validated to be associated with a significant change in enzyme activity or protein level (48, 49). So far, however, no research has been performed on analyzing the expressions, activities, and gene polymorphisms of UGT1A10 and SULT1E1 in GDM condition. Whether the elevation of serum E1-G/S concentration is related to the expressions or activities of the metabolic enzymes needs to be further confirmed, and the specific structures of the glucuronidation and sulfation metabolites changed in GDM need to be clarified. However, this study also has limitations, it is of great clinical significance to further confirm whether the steroid hormone indicators including 16OHE1 and E1-G/S exhibit similar changes in the first trimester of pregnancy, which may make the early prediction of GDM possible.

In summary, for the first time, the overall metabolic characteristics of steroid hormones in GDM women at second trimester during pregnancy were analyzed and summarized through quantifying 36 kinds of steroid hormones. Serum corticosteroids, progestins and especially the estrogen metabolites via 16-pathway from parent estrogens were higher in GDM women compared with healthy pregnant controls. Three estrogen-related markers, especially 16OHE1, may be closely associated with the risk for GDM. It is suggested that more attention should be paid to estrogen metabolism during pregnancy. Further research on the role of estrogen metabolites in the pathological process of GDM should be implemented.

## Data availability statement

The original contributions presented in the study are included in the article/**Supplementary Material**. Further inquiries can be directed to the corresponding authors.

## Ethics statement

The studies involving human participants were reviewed and approved by the ethical committee of Drum Tower Hospital Affiliated to Medical School of Nanjing University. The patients/participants provided their written informed consent to participate in this study.

## Author contributions

All authors participated in the interpretation of the study results and in the drafting, critical revision, and approval of the final version of the manuscript. NY, CJ, and HZ were involved in the study design and data analyses. JG and XZ were involved in subject recruitment and sample collection. JH, ML, and WZ were responsible for sample processing, data analyses and plotting. TZ, MW, and CJ were responsible for study monitoring. All authors contributed to the article.
