# Supplementary material for: Metabolic alteration of circulating steroid hormones in women with gestational diabetes mellitus and the related risk factors
Source: Front Endocrinol (Lausanne). 2023 Jun 15;14:1196935. doi: 10.3389/fendo.2023.1196935 (PMC10310992; doi:10.3389/fendo.2023.1196935)
Supplement: Supplementary file 1 [file DataSheet_1.docx]

Supplementary Material

Metabolic alteration of circulating steroid hormones in women with gestational diabetes mellitus and the related risk factors

Na Yang^1,2^†, Wei Zhang^3^†, Cheng Ji^1^†, Jiajia Ge^1^, Xiaoli Zhang^2^, Meijuan Li^3^, Min Wang^1^, Tianqi Zhang^1^, Jun He^2^*, Huaijun Zhu^1^*

^1^Department of Pharmacy, Nanjing Drum Tower Hospital, Afﬁliated Hospital of Medical School, Nanjing University, Nanjing 210008, Jiangsu, China

^2^Nanjing Drum Tower Hospital Clinical College of Nanjing University of Chinese Medicine, Nanjing 210023, Jiangsu, China

^3^Nanjing Qlife Medical Technology Co., Ltd, Nanjing 210032, Jiangsu, China

*** Correspondence:**Jun He
[hej@njucm.edu.cn](mailto:hej@njucm.edu.cn)
Huaijun Zhu
[huaijun.zhu@gmail.com](mailto:huaijun.zhu@gmail.com)
†These authors contributed equally to this work and share first authorship

# Supplementary Tables

**Table S1 The Calibration curves for each serum steroids**

| **Steroids** | **Linear equation** | **Correlation coefficient(r^2^)** |
| --- | --- | --- |
| **ALD** | Y=11.040446X+0.014427 | 0.9995 |
| **CORT** | Y=2.662968X+0.040123 | 0.9969 |
| **F** | Y=0.127629X+0.034795 | 0.9998 |
| **P4** | Y=2.402005X+0.030417 | 0.9998 |
| **17-OHP4** | Y=2.274118X+0.015482 | 0.9999 |
| **T** | Y=2.870377X+0.032739 | 0.9990 |
| **AD** | Y=2.002183X+0.029752 | 0.9998 |
| **DHT** | Y=8.381037X+0.000311 | 0.9988 |
| **DHEA** | Y=0.069479X-0.002275 | 0.9979 |
| **DHEAS** | Y=0.003371X+1.080038 | 0.9996 |
| **E1** | Y=0.0299019X+0.0042874 | 0.9999 |
| **E2** | Y=0.0396221X +0.0207631 | 0.9998 |
| **E3** | Y=0.00640514X+0.00216576 | 0.9997 |
| **16epiE3** | Y=0.00429246X+0.00139204 | 0.9989 |
| **17epiE3** | Y=0.00711493X+0.00613483 | 0.9994 |
| **2MeOE1** | Y=0.0531027X+0.00824421 | 0.9979 |
| **4MeOE1** | Y=0.0184119X-0.00426885 | 0.9994 |
| **16α-OHE1** | Y=0.00904973X+0.0369532 | 0.9995 |
| **2-OHE1** | Y=0.00377585X-0.00395876 | 0.9998 |
| **4-OHE1** | Y=0.00341747X-0.00343025 | 0.9985 |
| **2-OHE2** | Y=0.00342903X-0.00312112 | 0.9990 |

**Table S2 Accuracy and precision of the serum steroids(n=6)**

| **Steroids** | **Concentration(ng/mL)** | **RSD/%** | | **RE/%** |
| --- | --- | --- | --- | --- |
|  |  | **Intra-day(n=6)** | **Inter-day(n=18)** |  |
| **ALD** | 0.01 | 6.4 | 8.5 | 0.2 |
|  | 0.02 | 6.3 | 6.6 | 6.5 |
|  | 0.5 | 6.1 | 7.1 | 4.5 |
|  | 1.6 | 4.1 | 4.3 | 4.8 |
| **CORT** | 0.1 | 3.5 | 4.6 | -2.1 |
|  | 0.2 | 5.3 | 6.1 | 1.2 |
|  | 5.0 | 4.4 | 5.4 | 2.7 |
|  | 16.0 | 2.6 | 3.2 | -7.9 |
| **F** | 1.0 | 2.4 | 3.2 | -8.9 |
|  | 2.0 | 4.6 | 4.8 | 2.6 |
|  | 50.0 | 4.0 | 4.8 | 2.7 |
|  | 160.0 | 2.3 | 2.5 | 5.1 |
| **P4** | 0.05 | 4.0 | 4.8 | -0.8 |
|  | 0.1 | 8.6 | 9.1 | 1.1 |
|  | 2.5 | 8.7 | 6.0 | 4.0 |
|  | 8.0 | 2.5 | 1.8 | 4.4 |
| **17-OHP4** | 0.05 | 5.5 | 6.0 | -5.8 |
|  | 0.1 | 5.5 | 5.8 | 0.2 |
|  | 2.5 | 3.7 | 5.1 | 8.0 |
|  | 8.0 | 3.1 | 4.2 | 5.8 |
| **T** | 0.05 | 4.5 | 4.6 | -7.5 |
|  | 0.1 | 4.4 | 4.3 | 4.3 |
|  | 2.5 | 4.5 | 5.5 | 3.4 |
|  | 8.0 | 2.8 | 4.5 | 6.0 |
| **AD** | 0.05 | 3.3 | 3.5 | -0.1 |
|  | 0.1 | 3.9 | 3.8 | 1.6 |
|  | 2.5 | 4.4 | 5.7 | 5.8 |
|  | 8.0 | 1.5 | 1.9 | 4.4 |
| **DHT** | 0.025 | 9.3 | 9.1 | 3.2 |
|  | 0.05 | 8.4 | 8.7 | 1.6 |
|  | 1.25 | 7.1 | 7.4 | 3.0 |
|  | 4.0 | 5.5 | 6.0 | 5.7 |
| **DHEA** | 0.1 | 6.2 | 6.8 | -2.2 |
|  | 0.2 | 4.9 | 7.1 | 3.4 |
|  | 5.0 | 4.0 | 4.8 | 3.2 |
|  | 16.0 | 2.4 | 3.5 | 6.4 |
| **DHEAS** | 50 | 6.7 | 4.0 | 1.7 |
|  | 100.0 | 7.5 | 8.2 | 4.5 |
|  | 2500.0 | 5.5 | 5.8 | 3.2 |
|  | 8000.0 | 4.1 | 6.0 | 4.2 |
| **E1** | 1.0 | 2.9 | 5.5 | 1.2 |
|  | 2.0 | 6.1 | 9.2 | 2.8 |
|  | 50.0 | 5.7 | 5.5 | 3.4 |
|  | 400.0 | 3.0 | 3.2 | 3.9 |
| **E2** | 1.0 | 4.8 | 5.7 | -1.4 |
|  | 2.0 | 6.4 | 6.6 | 5.9 |
|  | 50.0 | 6.5 | 6.5 | 1.5 |
|  | 400.0 | 2.6 | 2.9 | 3.1 |
| **E3** | 1.0 | 5.1 | 5.2 | -3.9 |
|  | 2.0 | 8.2 | 8.8 | 1.9 |
|  | 50.0 | 5.0 | 5.5 | 0.2 |
|  | 400.0 | 2.6 | 3.0 | 2.4 |
| **16OHE1** | 1.0 | 7.8 | 8.3 | 0.0 |
|  | 2.0 | 8.9 | 8.6 | 2.0 |
|  | 50.0 | 3.6 | 5.1 | 4.3 |
|  | 400.0 | 2.3 | 4.0 | 2.5 |
| **16EpiE3** | 1.0 | 3.2 | 3.6 | -2.1 |
|  | 2.0 | 5.2 | 8.2 | 0.0 |
|  | 50.0 | 5.5 | 6.4 | 2.2 |
|  | 400.0 | 5.0 | 7.3 | 3.7 |
| **17EpiE3** | 1.0 | 5.3 | 5.3 | -1.6 |
|  | 2.0 | 3.6 | 7.2 | 2.0 |
|  | 50.0 | 5.2 | 7.5 | 0.9 |
|  | 400.0 | 5.1 | 7.3 | 2.8 |
| **2OHE1** | 1.0 | 3.5 | 5.3 | 2.7 |
|  | 2.0 | 7.8 | 8.6 | 0.5 |
|  | 50.0 | 4.8 | 4.8 | 9.5 |
|  | 400.0 | 5.8 | 7.7 | 0.1 |
| **2OHE2** | 1.0 | 6.3 | 6.6 | -1.0 |
|  | 2.0 | 8.5 | 9.7 | 0.7 |
|  | 50.0 | 6.6 | 9.8 | 4.0 |
|  | 400.0 | 7.2 | 8.5 | 2.6 |
| **2MeOE1** | 1.0 | 4.8 | 6.6 | 4.1 |
|  | 2.0 | 6.3 | 6.0 | 5.0 |
|  | 50.0 | 7.3 | 7.8 | 2.3 |
|  | 400.0 | 5.8 | 7.7 | 0.8 |
| **2MeOE2** | 1.0 | 5.6 | 8.3 | 0.5 |
|  | 2.0 | 7.4 | 7.7 | 2.4 |
|  | 50.0 | 5.8 | 7.8 | 0.3 |
|  | 400.0 | 4.8 | 7.1 | 0.3 |
| **4OHE1** | 1.0 | 5.2 | 6.5 | -0.9 |
|  | 2.0 | 7.3 | 7.3 | 2.4 |
|  | 50.0 | 3.2 | 5.1 | 10.6 |
|  | 400.0 | 6.0 | 6.4 | 5.1 |
| **4MeOE1** | 1.0 | 3.6 | 6.9 | 3.4 |
|  | 2.0 | 5.6 | 7.3 | 3.3 |
|  | 50.0 | 6.1 | 6.3 | 3.7 |
|  | 400.0 | 3.9 | 4.2 | 1.1 |
| **4MeOE2** | 1.0 | 2.9 | 9.3 | 1.3 |
|  | 2.0 | 10.3 | 13.3 | 0.2 |
|  | 50.0 | 5.7 | 6.3 | 0.9 |
|  | 400.0 | 4.8 | 4.9 | 0.1 |

**Table S3 Recovery rates and matrix effects of the serum steroids(‾X±SD)**

| **Steroids** | **Concentration(ng/mL)** | **Extraction recoveries /%**  **(n=3)** | **IS corrected matrix factors /%**  **(n=6)** |
| --- | --- | --- | --- |
| **ALD** | 0.02 | 61.1±4.9 | 114.3±12.6 |
|  | 0.5 | 61.7±4.0 | 93.0±3.3 |
|  | 1.6 | 69.6±1.8 | 102.2±5.9 |
| **CORT** | 0.2 | 149.0±6.2 | 113.7±2.6 |
|  | 5.0 | 164.3±1.9 | 92.2±2.4 |
|  | 16.0 | 151.0±2.3 | 100.7±3.8 |
| **F** | 2.0 | 127.7±3.0 | 125.3±4.5 |
|  | 50.0 | 125.1±0.3 | 92.7±2.7 |
|  | 160.0 | 104.8±4.4 | 99.3±4.2 |
| **P4** | 0.1 | 160.8±4.2 | 124.2±7.1 |
|  | 2.5 | 171.8±2.7 | 103.8±2.2 |
|  | 8.0 | 153.3±6.4 | 108.7±5.9 |
| **17-OHP4** | 0.1 | 158.0±7.3 | 123.7±5.8 |
|  | 2.5 | 182.7±4.4 | 94.7±2.7 |
|  | 8.0 | 158.1±4.0 | 101.5±4.9 |
| **T** | 0.1 | 160.8±4.8 | 115.0±3.2 |
|  | 2.5 | 183.4±1.4 | 94.7±2.1 |
|  | 8.0 | 163.0±3.0 | 100.8±4.5 |
| **AD** | 0.1 | 160.1±7.8 | 119.2±4.1 |
|  | 2.5 | 181.8±2.4 | 94.2±2.2 |
|  | 8.0 | 162.9±3.0 | 101.5±5.0 |
| **DHT** | 0.05 | 157.7±1.3 | 131.0±4.0 |
|  | 1.25 | 169.2±2.4 | 97.0±5.3 |
|  | 4.0 | 151.2±1.2 | 103.5±9.3 |
| **DHEA** | 0.2 | 130.0±9.1 | 98.2±9.0 |
|  | 5.0 | 122.5±0.4 | 94.8±5.0 |
|  | 16.0 | 99.2±6.0 | 99.0±7.6 |
| **DHEAS** | 100.0 | 0.8±0.0 | 111.2±1.5 |
|  | 2500.0 | 0.8±0.0 | 91.3±2.1 |
|  | 8000.0 | 0.7±0.0 | 99.3±3.6 |
| **E1** | 2.0 | 61.1±4.9 | 101.5±3.3 |
|  | 50.0 | 61.7±4.0 | 108.7±2.5 |
|  | 400.0 | 69.6±1.8 | 101.8±5.6 |
| **E2** | 2.0 | 69.1±2.2 | 100.8±4.4 |
|  | 50.0 | 65.1±2.9 | 100.7±1.0 |
|  | 400.0 | 74.5±2.6 | 96.2±5.2 |
| **E3** | 2.0 | 76.4±9.2 | 103.3±2.4 |
|  | 50.0 | 69.3±2.4 | 91.5±2.4 |
|  | 400.0 | 79.4±1.7 | 95.7±5.7 |
| **16OHE1** | 2.0 | 88.9±5.2 | 100.2±5.8 |
|  | 50.0 | 70.7±1.2 | 102.3±4.2 |
|  | 400.0 | 75.2±4.2 | 98.0±6.4 |
| **16EpiE3** | 2.0 | 74.1±5.5 | 99.5±2.6 |
|  | 50.0 | 74.4±2.0 | 100.0±6.1 |
|  | 400.0 | 78.5±1.5 | 93.0±6.2 |
| **17EpiE3** | 2.0 | 66.9±8.2 | 92.5±5.4 |
|  | 50.0 | 72.5±2.1 | 91.8±6.9 |
|  | 400.0 | 79.1±3.2 | 95.2±6.9 |
| **2OHE1** | 2.0 | 15.4±0.1 | 123.2±9.2 |
|  | 50.0 | 17.0±0.9 | 122.3±6.9 |
|  | 400.0 | 18.6±0.7 | 113.2±6.4 |
| **2OHE2** | 2.0 | 14.1±1.3 | 101.3±3.7 |
|  | 50.0 | 15.2±1.3 | 110.0±3.4 |
|  | 400.0 | 12.0±0.5 | 94.2±4.7 |
| **2MeOE1** | 2.0 | 84.9±0.7 | 68.3±3.7 |
|  | 50.0 | 80.2±3.8 | 68.2±5.4 |
|  | 400.0 | 93.4±5.7 | 87.3±2.0 |
| **2MeOE2** | 2.0 | 91.4±5.9 | 123.2±9.2 |
|  | 50.0 | 79.0±3.7 | 122.3±6.9 |
|  | 400.0 | 91.7±4.1 | 113.2±6.4 |
| **4OHE1** | 2.0 | 7.8±0.9 | 103.0±9.7 |
|  | 50.0 | 7.9±0.5 | 101.0±4.1 |
|  | 400.0 | 6.5±0.3 | 95.2±4.5 |
| **4MeOE1** | 2.0 | 50.9±5.2 | 104.0±2.1 |
|  | 50.0 | 52.2±3.9 | 120.2±4.8 |
|  | 400.0 | 59.2±1.0 | 106.8±5.8 |
| **4MeOE2** | 2.0 | 32.6±1.8 | 107.8±3.9 |
|  | 50.0 | 31.4±2.6 | 119.8±4.6 |
|  | 400.0 | 40.1±1.4 | 103.5±3.0 |

**Table S4. Stability evaluation of the serum steroids under different conditions (n=3)**

| **Steroids** | **Conditions** | **Concentrations(ng/ml)** | |  |  |
| --- | --- | --- | --- | --- | --- |
|  |  | **QC levels** | **Measurements** | **RSD/%** | **RE/%** |
| **ALD** | 6h at room | 0.02 | 0.02 | 11.1 | 5.9 |
|  |  | 0.5 | 0.5 | 0.8 | 1.2 |
|  |  | 1.6 | 1.51 | 1.5 | 2.1 |
|  | 24h at 4°C | 0.02 | 0.02 | 11.1 | 4.1 |
|  |  | 0.5 | 0.5 | 4.8 | 1.0 |
|  |  | 1.6 | 1.57 | 2.9 | 1.9 |
|  | 24h in autosampler at 8°C(Post-preparation) | 0.02 | 0.02 | 5.3 | 3.3 |
|  |  | 0.5 | 0.47 | 1.9 | 1.3 |
|  |  | 1.6 | 1.48 | 3.0 | 0.5 |
|  | Three freeze-thaw | 0.02 | 0.02 | 5.6 | 3.2 |
|  |  | 0.5 | 0.5 | 1.8 | 0.5 |
|  |  | 1.6 | 1.61 | 5.7 | 3.2 |
| **CORT** | 6h at room | 0.2 | 0.21 | 0.5 | 3.2 |
|  |  | 5.0 | 5.07 | 0.5 | 2.9 |
|  |  | 16.0 | 14.8 | 1.0 | 1.5 |
|  | 24h at 4°C | 0.2 | 0.2 | 3.0 | 1.9 |
|  |  | 5.0 | 5.07 | 0.7 | 3.0 |
|  |  | 16.0 | 15.26 | 0.6 | 1.5 |
|  | 24h in autosampler at 8°C(Post-preparation) | 0.2 | 0.2 | 4.5 | 6.2 |
|  |  | 5.0 | 4.69 | 1.2 | 1.9 |
|  |  | 16.0 | 14.39 | 1.5 | 0.8 |
|  | Three freeze-thaw | 0.2 | 0.2 | 2.0 | 3.0 |
|  |  | 5.0 | 5.1 | 0.3 | 2.1 |
|  |  | 16.0 | 15.51 | 1.5 | 3.3 |
| **F** | 6h at room | 2.0 | 1.98 | 0.7 | 1.8 |
|  |  | 50.0 | 49.13 | 0.5 | 2.1 |
|  |  | 160.0 | 149.32 | 0.8 | 2.9 |
|  | 24h at 4°C | 2.0 | 1.99 | 0.8 | 2.3 |
|  |  | 50.0 | 49.57 | 0.4 | 3.0 |
|  |  | 160.0 | 154.33 | 0.7 | 0.3 |
|  | 24h in autosampler at 8°C(Post-preparation) | 2.0 | 1.96 | 6.2 | 3.6 |
|  |  | 50.0 | 47.9 | 2.1 | 0.7 |
|  |  | 160.0 | 151.08 | 1.9 | 0.1 |
|  | Three freeze-thaw | 2.0 | 1.98 | 0.2 | 1.5 |
|  |  | 50.0 | 49.98 | 1.5 | 2.2 |
|  |  | 160.0 | 156.56 | 0.1 | 2.8 |
| **P4** | 6h at room | 0.1 | 0.1 | 4.2 | 2.4 |
|  |  | 2.5 | 2.53 | 0.6 | 3.6 |
|  |  | 8.0 | 7.7 | 0.5 | 4.1 |
|  | 24h at 4°C | 0.1 | 0.1 | 3.0 | 2.6 |
|  |  | 2.5 | 2.53 | 1.3 | 3.8 |
|  |  | 8.0 | 7.95 | 0.7 | 1.0 |
|  | 24h in autosampler at 8°C(Post-preparation) | 0.1 | 0.1 | 5.2 | 5.8 |
|  |  | 2.5 | 2.42 | 2.1 | 2.6 |
|  |  | 8.0 | 7.73 | 0.6 | 1.4 |
|  | Three freeze-thaw | 0.1 | 0.1 | 2.1 | 4.5 |
|  |  | 2.5 | 2.58 | 0.2 | 1.5 |
|  |  | 8.0 | 7.99 | 1.0 | 2.2 |
| **17-OHP4** | 6h at room | 0.1 | 0.09 | 2.1 | 3.1 |
|  |  | 2.5 | 2.41 | 1.3 | 4.5 |
|  |  | 8.0 | 7.65 | 2.1 | 1.2 |
|  | 24h at 4°C | 0.1 | 0.1 | 3.1 | 7.0 |
|  |  | 2.5 | 2.36 | 1.3 | 2.2 |
|  |  | 8.0 | 7.84 | 3.2 | 1.2 |
|  | 24h in autosampler at 8°C(Post-preparation) | 0.1 | 0.1 | 10.1 | 4.0 |
|  |  | 2.5 | 2.27 | 1.4 | 3.6 |
|  |  | 8.0 | 7.66 | 1.2 | 5.2 |
|  | Three freeze-thaw | 0.1 | 0.09 | 1.1 | 1.8 |
|  |  | 2.5 | 2.39 | 0.5 | 2.8 |
|  |  | 8.0 | 7.75 | 0.6 | 1.0 |
| **T** | 6h at room | 0.1 | 0.1 | 1.0 | 3.0 |
|  |  | 2.5 | 2.49 | 0.8 | 1.3 |
|  |  | 8.0 | 7.56 | 0.6 | 2.9 |
|  | 24h at 4°C | 0.1 | 0.1 | 1.0 | 1.3 |
|  |  | 2.5 | 2.52 | 0.7 | 2.7 |
|  |  | 8.0 | 7.8 | 0.5 | 0.2 |
|  | 24h in autosampler at 8°C(Post-preparation) | 0.1 | 0.09 | 5.4 | 3.0 |
|  |  | 2.5 | 2.4 | 2.8 | 3.2 |
|  |  | 8.0 | 7.69 | 0.9 | 7.1 |
|  | Three freeze-thaw | 0.1 | 0.1 | 3.2 | 1.8 |
|  |  | 2.5 | 2.56 | 1.0 | 1.8 |
|  |  | 8.0 | 7.8 | 1.0 | 1.9 |
| **AD** | 6h at room | 0.1 | 0.1 | 2.9 | 4.9 |
|  |  | 2.5 | 2.51 | 0.4 | 5.4 |
|  |  | 8.0 | 7.88 | 0.3 | 1.8 |
|  | 24h at 4°C | 0.1 | 0.1 | 1.0 | 7.0 |
|  |  | 2.5 | 2.52 | 0.7 | 6.1 |
|  |  | 8.0 | 8.14 | 0.6 | 5.2 |
|  | 24h in autosampler at 8°C(Post-preparation) | 0.1 | 0.1 | 10.1 | 1.6 |
|  |  | 2.5 | 2.37 | 1.1 | 4.8 |
|  |  | 8.0 | 7.74 | 1.2 | 2.7 |
|  | Three freeze-thaw | 0.1 | 0.1 | 2.1 | 1.7 |
|  |  | 2.5 | 2.46 | 1.4 | 2.0 |
|  |  | 8.0 | 7.79 | 0.4 | 1.6 |
| **DHT** | 6h at room | 0.05 | 0.05 | 1.9 | 5.3 |
|  |  | 1.25 | 1.2 | 1.3 | 6.3 |
|  |  | 4.0 | 3.68 | 4.1 | 10.1 |
|  | 24h at 4°C | 0.05 | 0.05 | 15.0 | 4.2 |
|  |  | 1.25 | 1.22 | 6.9 | 4.4 |
|  |  | 4.0 | 3.57 | 3.7 | 12.7 |
|  | 24h in autosampler at 8°C(Post-preparation) | 0.05 | 0.05 | 5.7 | 1.8 |
|  |  | 1.25 | 1.18 | 4.3 | 1.6 |
|  |  | 4.0 | 3.77 | 1.4 | 2.3 |
|  | Three freeze-thaw | 0.05 | 0.05 | 3.8 | 9.6 |
|  |  | 1.25 | 1.31 | 5.3 | 4.7 |
|  |  | 4.0 | 3.78 | 0.3 | 2.8 |
| **DHEA** | 6h at room | 0.2 | 0.18 | 4.4 | 0.2 |
|  |  | 5.0 | 4.99 | 0.6 | 4.4 |
|  |  | 16.0 | 14.57 | 2.2 | 3.1 |
|  | 24h at 4°C | 0.2 | 0.18 | 1.7 | 1.2 |
|  |  | 5.0 | 4.82 | 1.2 | 1.0 |
|  |  | 16.0 | 14.98 | 2.4 | 0.3 |
|  | 24h in autosampler at 8°C(Post-preparation) | 0.2 | 0.18 | 2.2 | 11.0 |
|  |  | 5.0 | 4.5 | 1.8 | 6.4 |
|  |  | 16.0 | 14.59 | 4.3 | 0.6 |
|  | Three freeze-thaw | 0.2 | 0.18 | 6.5 | 4.8 |
|  |  | 5.0 | 4.87 | 1.2 | 3.5 |
|  |  | 16.0 | 15.21 | 2.2 | 3.0 |
| **DHEAS** | 6h at room | 100.0 | 65.63 | 4.1 | 4.9 |
|  |  | 2500.0 | 2308.81 | 1.1 | 0.0 |
|  |  | 8000.0 | 7216.07 | 0.6 | 3.0 |
|  | 24h at 4°C | 100.0 | 83.14 | 3.0 | 13.4 |
|  |  | 2500.0 | 2356.01 | 1.7 | 2.9 |
|  |  | 8000.0 | 7426.85 | 3.8 | 1.4 |
|  | 24h in autosampler at 8°C(Post-preparation) | 100.0 | 109.15 | 5.2 | 13.8 |
|  |  | 2500.0 | 2464.95 | 5.6 | 1.6 |
|  |  | 8000.0 | 8040.12 | 2.3 | 6.7 |
|  | Three freeze-thaw | 100.0 | 55.3 | 4.0 | 9.3 |
|  |  | 2500.0 | 2322.82 | 1.8 | 0.7 |
|  |  | 8000.0 | 7521.52 | 1.5 | 4.8 |
| **E1** | 2h at room | 2.0 | 1.81 | 3.2 | 7.3 |
|  |  | 50.0 | 49.48 | 0.4 | 5.3 |
|  |  | 400.0 | 401.56 | 0.6 | 1.6 |
|  | 12h at 4°C | 2.0 | 1.96 | 8.1 | 0.6 |
|  |  | 50.0 | 48.38 | 0.7 | 2.9 |
|  |  | 400.0 | 428.44 | 0.5 | 5.0 |
|  | 24h in autosampler at 8°C(Post-preparation) | 2.0 | 1.84 | 1.1 | 5.5 |
|  |  | 50.0 | 47.45 | 5.1 | 0.9 |
|  |  | 400.0 | 359.61 | 4.6 | 11.8 |
|  | Three freeze-thaw | 2.0 | 1.97 | 1.3 | 0.8 |
|  |  | 50.0 | 49.65 | 1.9 | 5.6 |
|  |  | 400.0 | 368.43 | 2.1 | 9.7 |
| **E2** | 2h at room | 2.0 | 2.19 | 3.8 | 14.7 |
|  |  | 50.0 | 46.31 | 1.4 | 11.1 |
|  |  | 400.0 | 415.56 | 0.9 | 5.5 |
|  | 12h at 4°C | 2.0 | 2.22 | 1.4 | 14.7 |
|  |  | 50.0 | 44.69 | 0.4 | 11.1 |
|  |  | 400.0 | 412.52 | 0.3 | 5.5 |
|  | 24h in autosampler at 8°C(Post-preparation) | 2.0 | 2.21 | 0.5 | 14.5 |
|  |  | 50.0 | 46.24 | 1.4 | 8.0 |
|  |  | 400.0 | 361.64 | 2.7 | 7.5 |
|  | Three freeze-thaw | 2.0 | 1.96 | 5.6 | 1.7 |
|  |  | 50.0 | 49.74 | 3.0 | 1.1 |
|  |  | 400.0 | 348.74 | 0.7 | 10.8 |
| **E3** | 2h at room | 2.0 | 1.91 | 3.2 | 0.1 |
|  |  | 50.0 | 51.4 | 2.1 | 10.3 |
|  |  | 400.0 | 336.15 | 1.1 | 10.1 |
|  | 12h at 4°C | 2.0 | 1.99 | 2.5 | 4.2 |
|  |  | 50.0 | 47.38 | 0.6 | 1.7 |
|  |  | 400.0 | 359.91 | 0.6 | 3.7 |
|  | 24h in autosampler at 8°C(Post-preparation) | 2.0 | 1.85 | 4.2 | 3.1 |
|  |  | 50.0 | 44.96 | 2.1 | 3.5 |
|  |  | 400.0 | 364.28 | 12.0 | 2.6 |
|  | Three freeze-thaw | 2.0 | 2.02 | 4.6 | 6.2 |
|  |  | 50.0 | 52.36 | 2.8 | 12.3 |
|  |  | 400.0 | 349.84 | 0.3 | 6.4 |
| **16OHE1** | 2h at room | 2.0 | 2.04 | 6.2 | 2.7 |
|  |  | 50.0 | 48.86 | 2.2 | 1.1 |
|  |  | 400.0 | 358.57 | 3.2 | 5.7 |
|  | 12h at 4°C | 2.0 | 2.04 | 1.2 | 2.8 |
|  |  | 50.0 | 47.99 | 0.5 | 2.9 |
|  |  | 400.0 | 366.19 | 1.0 | 3.7 |
|  | 24h in autosampler at 8°C(Post-preparation) | 2.0 | 1.9 | 4.1 | 4.3 |
|  |  | 50.0 | 46.92 | 0.6 | 5.0 |
|  |  | 400.0 | 366.15 | 9.3 | 3.7 |
|  | Three freeze-thaw | 2.0 | 2.01 | 4.7 | 1.3 |
|  |  | 50.0 | 52.8 | 0.4 | 6.9 |
|  |  | 400.0 | 371.55 | 4.9 | 2.3 |
| **16EpiE3** | 2h at room | 2.0 | 2.02 | 6.4 | 2.8 |
|  |  | 50.0 | 47.67 | 2.8 | 5.5 |
|  |  | 400.0 | 353.23 | 1.7 | 0.3 |
|  | 12h at 4°C | 2.0 | 2 | 5.6 | 1.6 |
|  |  | 50.0 | 49.31 | 1.3 | 2.2 |
|  |  | 400.0 | 343.12 | 1.5 | 3.1 |
|  | 24h in autosampler at 8°C(Post-preparation) | 2.0 | 1.99 | 5.3 | 1.0 |
|  |  | 50.0 | 52.54 | 1.9 | 4.2 |
|  |  | 400.0 | 376.45 | 4.1 | 6.3 |
|  | Three freeze-thaw | 2.0 | 1.89 | 3.6 | 3.8 |
|  |  | 50.0 | 52.81 | 2.3 | 4.8 |
|  |  | 400.0 | 349.1 | 4.0 | 1.5 |
| **17EpiE3** | 2h at room | 2.0 | 1.99 | 3.5 | 1.0 |
|  |  | 50.0 | 48.87 | 2.5 | 2.8 |
|  |  | 400.0 | 371.85 | 1.8 | 1.6 |
|  | 12h at 4°C | 2.0 | 2.01 | 3.5 | 0.2 |
|  |  | 50.0 | 49.64 | 2.2 | 1.3 |
|  |  | 400.0 | 361.91 | 0.5 | 1.1 |
|  | 24h in autosampler at 8°C(Post-preparation) | 2.0 | 1.98 | 4.7 | 1.7 |
|  |  | 50.0 | 53.15 | 5.5 | 5.7 |
|  |  | 400.0 | 405.84 | 5.6 | 10.9 |
|  | Three freeze-thaw | 2.0 | 1.97 | 2.8 | 1.9 |
|  |  | 50.0 | 54.09 | 2.1 | 7.6 |
|  |  | 400.0 | 361.98 | 2.9 | 1.1 |
| **2OHE1** | 2h at room | 2.0 | 1.89 | 4.2 | 0.1 |
|  |  | 50.0 | 47.07 | 1.2 | 4.0 |
|  |  | 400.0 | 363.23 | 4.1 | 3.7 |
|  | 12h at 4°C | 2.0 | 1.94 | 6.1 | 3.0 |
|  |  | 50.0 | 48.59 | 1.0 | 0.9 |
|  |  | 400.0 | 374.91 | 1.3 | 0.6 |
|  | 24h in autosampler at 8°C(Post-preparation) | 2.0 | 2.03 | 3.3 | 7.6 |
|  |  | 50.0 | 49.36 | 5.4 | 0.7 |
|  |  | 400.0 | 394.42 | 15.2 | 4.6 |
|  | Three freeze-thaw | 2.0 | 2.01 | 4.4 | 6.8 |
|  |  | 50.0 | 51.35 | 5.7 | 4.7 |
|  |  | 400.0 | 365.55 | 2.9 | 3.0 |
| **2OHE2** | 2h at room | 2.0 | 1.88 | 5.5 | 1.0 |
|  |  | 50.0 | 45.68 | 2.1 | 7.8 |
|  |  | 400.0 | 365.17 | 6.7 | 4.2 |
|  | 12h at 4°C | 2.0 | 1.96 | 2.4 | 5.6 |
|  |  | 50.0 | 53.64 | 0.6 | 8.3 |
|  |  | 400.0 | 394.92 | 0.8 | 3.6 |
|  | 24h in autosampler at 8°C(Post-preparation) | 2.0 | 1.87 | 7.5 | 0.7 |
|  |  | 50.0 | 48.67 | 9.1 | 1.8 |
|  |  | 400.0 | 383.35 | 12.8 | 0.6 |
|  | Three freeze-thaw | 2.0 | 2.07 | 6.8 | 11.4 |
|  |  | 50.0 | 56.22 | 4.6 | 13.5 |
|  |  | 400.0 | 417.36 | 3.2 | 9.5 |
| **2MeOE1** | 2h at room | 2.0 | 2 | 5.7 | 3.1 |
|  |  | 50.0 | 46.97 | 1.9 | 9.7 |
|  |  | 400.0 | 406.95 | 3.1 | 3.3 |
|  | 12h at 4°C | 2.0 | 2.04 | 4.4 | 1.1 |
|  |  | 50.0 | 49.7 | 0.4 | 4.5 |
|  |  | 400.0 | 425.84 | 1.4 | 1.2 |
|  | 24h in autosampler at 8°C(Post-preparation) | 2.0 | 2.06 | 2.1 | 0.1 |
|  |  | 50.0 | 49.06 | 4.6 | 5.7 |
|  |  | 400.0 | 399.99 | 4.0 | 4.9 |
|  | Three freeze-thaw | 2.0 | 2.24 | 1.3 | 8.5 |
|  |  | 50.0 | 56.79 | 3.0 | 9.2 |
|  |  | 400.0 | 457.62 | 3.9 | 8.8 |
| **2MeOE2** | 2h at room | 2.0 | 1.96 | 2.9 | 3.5 |
|  |  | 50.0 | 45.19 | 1.7 | 8.3 |
|  |  | 400.0 | 346.25 | 1.5 | 7.4 |
|  | 12h at 4°C | 2.0 | 2.03 | 4.1 | 7.0 |
|  |  | 50.0 | 47.92 | 0.3 | 2.8 |
|  |  | 400.0 | 364.3 | 1.1 | 2.5 |
|  | 24h in autosampler at 8°C(Post-preparation) | 2.0 | 1.89 | 2.1 | 0.0 |
|  |  | 50.0 | 49.01 | 8.4 | 0.5 |
|  |  | 400.0 | 383.47 | 15.2 | 2.6 |
|  | Three freeze-thaw | 2.0 | 2.17 | 3.6 | 14.6 |
|  |  | 50.0 | 54.8 | 2.9 | 11.2 |
|  |  | 400.0 | 399.61 | 1.1 | 6.9 |
| **4OHE1** | 2h at room | 2.0 | 1.84 | 3.4 | 1.8 |
|  |  | 50.0 | 50.15 | 0.8 | 5.5 |
|  |  | 400.0 | 387.91 | 1.3 | 1.8 |
|  | 12h at 4°C | 2.0 | 1.98 | 7.7 | 5.7 |
|  |  | 50.0 | 53.05 | 0.4 | 0.1 |
|  |  | 400.0 | 404.47 | 2.0 | 6.1 |
|  | 24h in autosampler at 8°C(Post-preparation) | 2.0 | 1.94 | 8.8 | 3.3 |
|  |  | 50.0 | 49.81 | 5.4 | 6.2 |
|  |  | 400.0 | 398.41 | 15.0 | 4.6 |
|  | Three freeze-thaw | 2.0 | 1.92 | 5.5 | 2.6 |
|  |  | 50.0 | 57.43 | 3.9 | 8.2 |
|  |  | 400.0 | 392.81 | 0.7 | 3.1 |
| **4MeOE1** | 2h at room | 2.0 | 1.95 | 1.1 | 2.3 |
|  |  | 50.0 | 47.22 | 1.8 | 3.2 |
|  |  | 400.0 | 391.52 | 2.6 | 0.1 |
|  | 12h at 4°C | 2.0 | 1.99 | 7.4 | 0.4 |
|  |  | 50.0 | 48.79 | 0.3 | 0.1 |
|  |  | 400.0 | 424.91 | 0.2 | 8.7 |
|  | 24h in autosampler at 8°C(Post-preparation) | 2.0 | 1.98 | 7.0 | 0.7 |
|  |  | 50.0 | 49.77 | 8.0 | 2.1 |
|  |  | 400.0 | 355.47 | 4.0 | 9.1 |
|  | Three freeze-thaw | 2.0 | 1.98 | 4.4 | 0.7 |
|  |  | 50.0 | 50.35 | 2.1 | 3.3 |
|  |  | 400.0 | 377.68 | 1.4 | 3.4 |
| **4MeOE2** | 2h at room | 2.0 | 2 | 4.1 | 2.1 |
|  |  | 50.0 | 48.03 | 1.8 | 4.9 |
|  |  | 400.0 | 406.79 | 0.9 | 3.1 |
|  | 12h at 4°C | 2.0 | 2.18 | 7.8 | 6.7 |
|  |  | 50.0 | 55.3 | 0.3 | 9.5 |
|  |  | 400.0 | 448.83 | 2.2 | 13.7 |
|  | 24h in autosampler at 8°C(Post-preparation) | 2.0 | 2.1 | 2.6 | 2.7 |
|  |  | 50.0 | 49.85 | 5.3 | 1.3 |
|  |  | 400.0 | 379.61 | 3.6 | 3.8 |
|  | Three freeze-thaw | 2.0 | 2.08 | 6.6 | 1.8 |
|  |  | 50.0 | 51.1 | 3.0 | 1.2 |
|  |  | 400.0 | 397.69 | 3.8 | 0.8 |
